# Magnetic resonance guided adaptive post prostatectomy radiotherapy: Accumulated dose comparison of different workflows

**DOI:** 10.1002/acm2.14253

**Published:** 2024-02-23

**Authors:** Sean P. Hassan, Jeremiah de Leon, Vikneswary Batumalai, Zoe Moutrie, Louise Hogan, Yuanyuan Ge, Phillip Stricker, Michael G. Jameson

**Affiliations:** ^1^ GenesisCare St Vincent's Hospital Sydney New South Wales Australia; ^2^ Faculty of Medicine University of New South Wales Sydney New South Wales Australia; ^3^ South Western Sydney Cancer Services New South Wales Health Sydney Australia; ^4^ Ingham Institute for Applied Medical Research Sydney Australia; ^5^ GenesisCare Murdoch Western Australia Australia; ^6^ Western Sydney University Penrith New South Wales Australia; ^7^ St Vincent's Prostate Cancer Research Centre Darlinghurst New South Wales Australia; ^8^ Garvan Institute, Darlinghurst Sydney New South Wales Australia; ^9^ University of Sydney Camperdown New South Wales Australia; ^10^ Centre for Medical Radiation Physics University of Wollongong Wollongong Australia

**Keywords:** adaptive radiotherapy, MRL, MR‐Linac, post‐prostatectomy radiotherapy, prostate cancer, radiotherapy

## Abstract

**Purpose:**

The aim of this study was to assess the use of magnetic resonance guided adaptive radiotherapy (MRgART) in the post‐prostatectomy setting; comparing dose accumulation for our initial seven patients treated with fully adaptive workflow on the Unity MR‐Linac (MRL) and with non‐adaptive plans generated offline. Additionally, we analyzed toxicity in patients receiving treatment.

**Methods:**

Seven patients were treated with MRgART. The prescription was 70–72 Gy in 35–36 fractions. Patients were treated with an adapt to shape (ATS) technique. For each clinically delivered plan, a non‐adaptive plan based upon the reference plan was generated and compared to the associated clinically delivered plan. A total of 468 plans were analyzed. Concordance Index of target and Organs at Risk (OARs) for each fraction with reference contours was analyzed. Acute toxicity was then assessed at six‐months following completion of treatment with Common Terminology for Adverse Events (CTCAE) Toxicity Criteria.

**Results:**

A total of 246 fractions were clinically delivered to seven patients; 234 fractions were delivered via MRgART and 12 fractions delivered via a traditional linear accelerator due to machine issues. Pre‐treatment reference plans met CTV and OAR criteria. PTV coverage satisfaction was higher in the clinically delivered adaptive plans than non‐adaptive comparison plans; 42.93% versus 7.27% respectively. Six‐month CTCAE genitourinary and gastrointestinal toxicity was absent in most patients, and mild‐to‐moderate in a minority of patients (Grade 1 GU toxicity in one patient and Grade 2 GI toxicity in one patient).

**Conclusions:**

Daily MRgART treatment consistently met planning criteria. Target volume variability in prostate bed treatment can be mitigated by using MRgART and deliver satisfactory coverage of CTV whilst minimizing dose to adjacent OARs and reducing toxicity

## 1 INTRODUCTION

Salvage prostate bed radiotherapy has been shown to improve biochemical free survival.^1^, ^2^ The clinical target volume (CTV) is defined anteriorly and posteriorly by the organs at risk (OARs) of the bladder and rectum respectively.^1,2^ Interfraction changes in the position and size of these OARs therefore impact the CTV.^3^ Further, compounding this is the difficulty both in delineating volumes on the initial planning CT and confirming consistency in shape, size and position of the volumes throughout treatment.^4^ This may potentially lead to geographical miss or violations of OAR constraints during treatment.^4^

The MR Linac (MRL) combines an MRI and Linear Accelerator allowing daily adaptive treatment based on soft tissue imaging and provides technology to account for these interfraction changes. This allows optimization of appropriate target volume coverage whilst minimizing dose to adjacent OARs.^5^ MRL planning on the Elekta Unity (Elekta Solutions AB, Stockholm, Sweden) with an inbuilt with a Philips Marlin 1.5 T MRI scanner (Philips Healthcare, Best, the Netherlands) is achieved by creating an initial offline (or reference) plan, and then, broadly, two adaption workflows can be applied for daily plan modification. These are: adapt to position (ATP) whereby a virtual isocenter shift is applied to account for patient positioning day‐to‐day and adapt to shape (ATS); a fully adaptive process consisting of recontouring and re‐optimization whilst the patient is on the treatment couch. Both workflows use MR images to allow improved soft tissue visualization and make use of automated contour propagation methods.^6^ The fully adaptive ATS method offers greater flexibility in accounting for inter‐fraction organ shape and positional variation but has been shown to extend daily treatment time.^7^, ^8^ Extended on bed time affects intra‐fractional uncertainty and can lead to target underdosage and any time saving efforts appear to offer a dosimetric advantage to MR guided online adaptive radiation therapy.^9^ Users have reported that increased on‐bed time in the ATS workflow compared to the ATP workflow attributed to the added step in‐contour generation and modification.^10^ The deformable registration algorithm employed for non‐rigid anatomical structures can lead to significant time editing of contours.^11^ Whilst a library of plans technique can provide an alternative to account for inter and intrafraction size and shape, its utility is often limited to when there is one dominant variable impacting a plan, such as bladder filling for adaptive bladder RT. When treating the prostate bed, the variability in bladder and bowel filling makes the sole reliance on a library impractical.

Deformable image registration (DIR) has gained clinical prominence in recent years through commercial availability.^12^, ^13^ The applications of DIR in radiotherapy include contour propagation, dose accumulation, and response assessment. One of the limitations on widespread uptake of DIR has been the difficulty in assessing the accuracy and uncertainty of the results.^13^ Dose accumulation can be a useful tool when assessing the value of different plan adaptation strategies. When the datasets used for DIR have been delineated by experts these contours may be used to assess the accuracy of the result or be incorporated in the algorithm as boundary conditions. The Dice similarity coefficient (DICE) may be used to assess the degree of agreement between contours and is widely used but does have limitations, such as scaling with volume.^14^

Magnetic resonance guided adaptive radiotherapy (MRgART) endeavors to provide better target volume coverage whilst minimizing dose to surrounding OARs. There are varying early evidence with some suggestion that there may not be significant change of OAR exposure with online plan adaption for intact prostate radiotherapy.^15^ However, given the interfraction variability of pelvic anatomy, adaptative radiotherapy could be useful for treatment of prostate bed. It is nevertheless resource intensive due to the number of fractions. For long course MRgRT in our clinic we have employed a hybrid adaption workflow by initiating an ATS workflow that uses perform adaption from a library of plans comprised of the offline/reference plan and first five fractions (created using ATS), which is intended to expedite the contour propagation and modification step of the ATS workflow. Using DIR, we compared adaptive and non‐adaptive plans to assess the value of MRgART for patients. We describe our initial clinical experience and report on the dosimetric comparison of ATS and conventional IMRT workflow on a 1.5 T MR Linac for the treatment of prostate bed radiotherapy. Previous work examining the use of MRgART in delivery stereotactic radiotherapy to the prostate bed has been undertaken,^16^ whereas the current study has examined its use to traditionally fractionated treatment where the ability to adapt for changes in anatomy over a full course of treatment. Further research exploring MRgART in prostate bed standard fractionation radiotherapy has examined the use of ATS and ATP as well as early toxicity data.^17^ To better understand both the impact of full treatment adaptation verses standard of care treatment, dose comparison through analysis of geometric and dosimetric data will further assist in determining the wider utility of MRgART.^16^

## 2 METHODS

### 2.1 Eligibility

Between October 2020 to June 2021, seven consecutive patients suitable for prostate bed radiotherapy were prospectively recruited to our Institutional Review Board approved ADAPT‐MRL registry.^18^ All patients had either a PSA of ≥0.2 ng/mL or three consecutive rises in PSA and Prostate Specific Membrane Antigen (PSMA) scans showing no evidence of distant disease. Patients with contraindications for MRI including claustrophobia or MRI non‐compatible artificial implants were excluded.

### 2.2 Simulation and reference plan generation

All patients underwent a planning MRI on the Elekta Unity MR Linac in addition to a planning CT scan (Siemens Somatom Definition AS, Siemens Healthineers, Erlangen, Germany) using our department protocol (120 kV, 2 mm slices). All patients followed a standardized departmental protocol for bowel and bladder preparation both for simulation and throughout treatment. Bowel preparation consisted of a modified diet and instruction to patients to empty their bowels prior to radiotherapy. Bladder preparation consisted of complete voiding of their bladder 30 min prior to their scan, then drinking 200 mL of water. A Verathon BVI‐640 bladder scanner ultrasound (Verathon, Bothell, WA, USA) was used to measure the urinary bladder volume at simulation and daily before each fraction. Due to an expected longer on‐bed time compared to conventional linear accelerator treatments, we chose this bladder preparation regime to balance appropriate filling and to manage patient comfort over the course of the fraction. Daily adaptation enabled us to compensate for any day‐to‐day variability.

At time 0 (*t*0) a MRI was carried out on the Unity using a 2‐min T2 scan. A second T2 scan is carried out at 25 min (*t*25) to assess bladder filling and rectal movement. This time point was chosen to reflect the average time from initial scan to beam on during treatment. If the key anatomy had moved such that the target volume was no longer covered by the PTV we would re‐image and perform ATP accounting for the organ drift.

The CTV and OARs were contoured as per Royal Australia and New Zealand College of Radiologists, Faculty of Radiation Oncology Genitourinary Group (RANZCR FROGG) guidelines on the *t*0 MRI sequence.^19^ The CTV to planning target volume (PTV) margin was 0.5 cm. These contours were then reviewed on the *t*25 MR image to assess target organ changes. Rectum, bladder and femoral heads were contoured as the OARs. Dose constraints followed RANZCR FROGG guidelines.^19^ Patients were planned to 70–72 Gy in 35–36 fractions treated daily.

### 2.3 Planning template

The planning template consisted of a 7 MV flattening filter free (FFF) intensity modulated radiotherapy (IMRT) plan with nine beams angles: 0, 50, 80, 120, 150, 210, 240, 280, and 310°. Planning was achieved using a grid resolution of 3 mm grid for both optimization and final dose calculation in Monaco (V6 Elekta Solutions AB, Stockholm, Sweden) treatment planning system, dose calculations were set to a statistical uncertainty of 1%. Optimization was performed using “optimize weights and shapes from fluence” over all voxels in volume, with fluence smoothing set to medium and maximum number of segments of 150^7^ Dose constraints were based upon published guidelines^19^ specifically, rectum V40Gy < 60%, V60Gy < 40%; as well as ensuring the 50% isodose did not cover the entire rectal circumference at any point, and bladder V50Gy < 50%.

### 2.4 Online adaptive workflow

**FIGURE 1:**
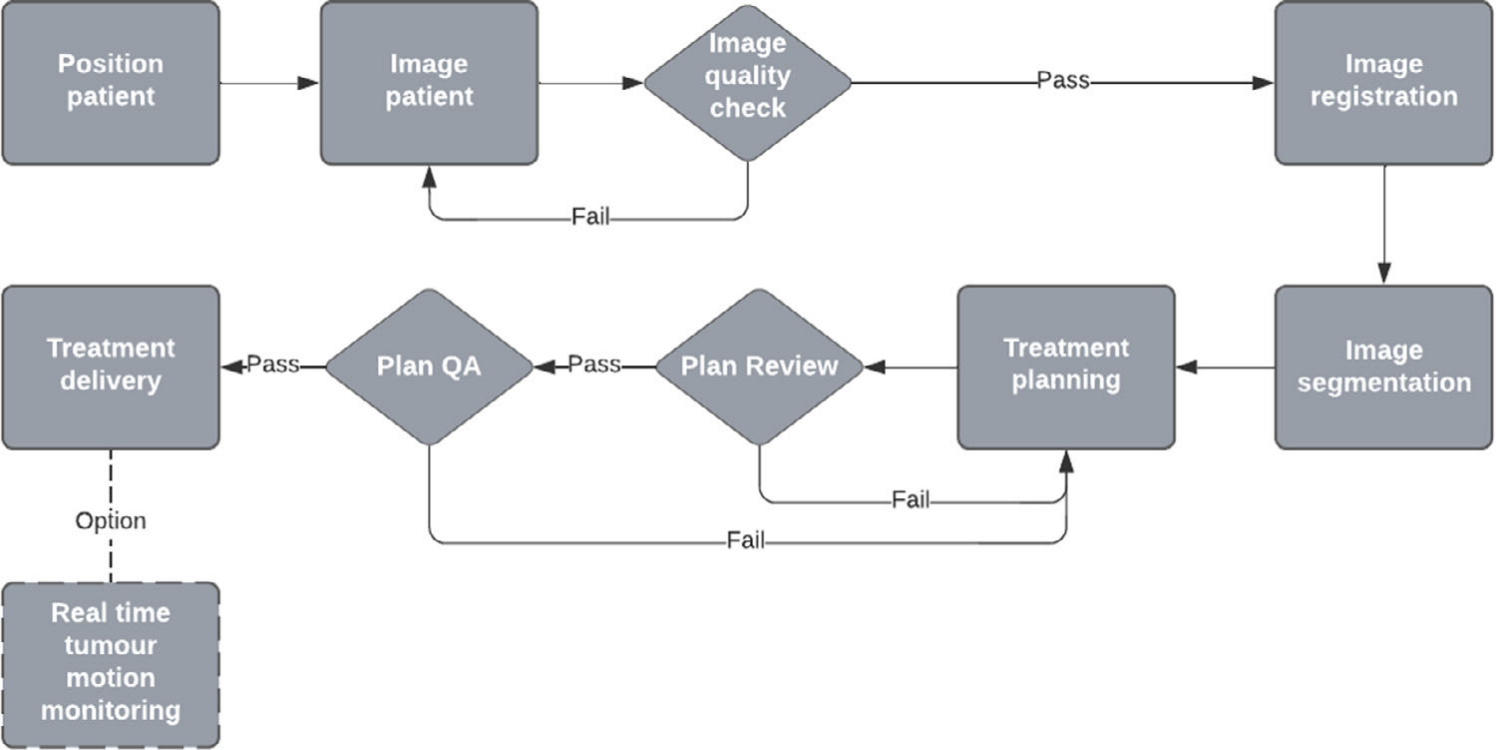
Daily online adaptive workflow for MRgART.

Patients followed the same bladder and bowel protocol as at simulation. A transverse T2 MRI sequence similar to that used at simulation was obtained and sent to Monaco. To reduce time spent editing or recontouring structures, the daily reference plan was chosen from a library of previous fractions, selecting that with the closest anatomy. For each patient, the initial five fractions were used to create a library of the patient's typical range of daily anatomy. The library consisted of the first five fractions of the treatment. For these fractions we documented key aspects of the anatomy such as volumes and proximity to adjacent organs as well as planning parameters with screen captures in an eScribe document in MOSAIQ (Elekta Solutions AB, Stockholm, Sweden). This document was then used to pick the most similar reference image and plan for the daily MRI acquired at >5 fractions. This was referred to at the image registration step to select the fraction with the most similar anatomy to the current fractions’ MRI (Figure 1). This allowed for daily variations in contours to efficiently and effectively propagate the contours online by matching to a library of anatomy. When using the ATS workflow, the optimization parameters and objectives were those that were set and approved at the reference planning stage outlined in Section 2.4. When moving from contouring to plan re‐optimization this was the starting point. For our workflow the reference plan was either initial plan or one of the 5 library plans from previous fractions.

Following image registration, when necessary, the external (body), CTV, rectum and bladder were re‐contoured by the Radiation Oncologist (RO). For each fraction, all patient plans underwent online treatment plan adaption using the ATS workflow. The radiotherapy plan created offline on the reference anatomy was re‐optimized and recalculated based on the anatomy of that fraction or a previous fraction. Plans were reviewed and approved by the RO. Offline reference plan patient specific pre‐treatment quality assurance consisted of independent monitor unit check performed using RadCalc (LifeLine Software, Inc, LAP Group, Tyler, Texas USA) and measurement‐based checks were performed using MRArcCheck (Sun Nuclear, Melbourne, FL USA) and ionization chamber point dose measurements in high dose region, using a 3%/3 mm gamma with 10% threshold, 95% passing rate and 2% tolerance for point dose. Online plan quality assurance consisted of using the traffic light system of plan goals in Monaco in addition to an independent monitor unit check using RadCalc. Additionally, post‐treatment patient specific quality assurance was performed on the first adapted fraction using the MRArcCheck and ionization chamber.

Prior to beam on, a Motion Management (MM) image was taken. This consisted of 2D MRI cine images (four frames‐per‐second, slice thickness 5 mm) in three planes taken at the centre of the PTV.^20^ This was used by the RO to visually assess for any significant movement or anatomical change prior to beam on.^21^

### 2.5 Dose comparison of different workflows

**FIGURE 2:**
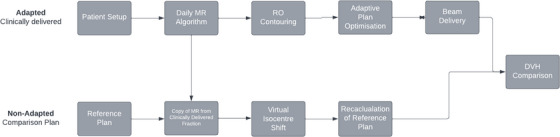
Comparison plan workflow.

All clinically delivered MRgART fractions were analyzed offline to compare accumulated dose using MRL workflows of ATS and compared to reference plan DVHs (Figure 2). Concordance Index of target and OARs for each fraction with reference contours were analyzed.

**FIGURE 3:**
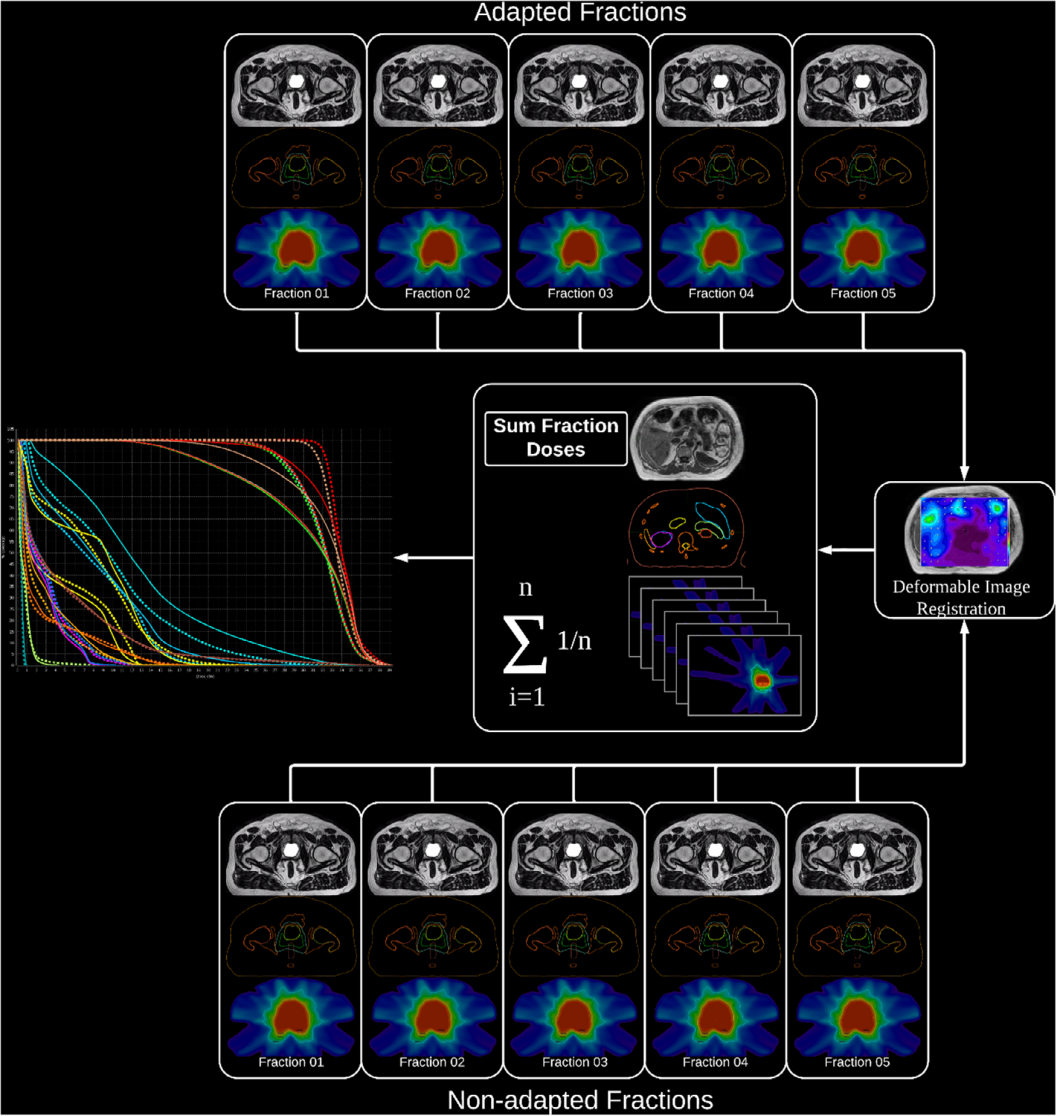
Deformable image registration workflow.

**FIGURE 4:**
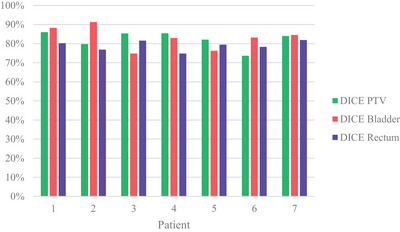
Mean DICE coefficients for PTV, bladder and rectum volumes.

Dose accumulation was performed in MIM (MIM Software Inc., Cleveland, OH, USA). All MRs were deformably registered to Fraction 1 using a constrained intensity‐based, free‐form deformable registration algorithm with a feature similarity scoring metric. This algorithm is a general‐use multi‐modality free‐form deformation that uses diffusion regularization and a feature similarity scoring metric. It aims to maximize the correspondence of high‐dimensional feature descriptors computed by evaluating each image voxel in the context of its neighboring voxels.^22^ The generated deformations were used to put all dose cubes into the fraction one frame of reference where they were summed (Figure 3). The DICE similarity coefficient was calculated for the bladder, rectum and PTV to assess the accuracy of deformable image registration (Figure 4).

### 2.6 Acute toxicity

Patient toxicity was evaluated by the treating Radiation Oncologist at three‐ and six‐months post completion of radiotherapy utilizing Common Terminology Criteria for Adverse Events (CTCAE) Grading Scheme.^23^

## 3 RESULTS

Seven patients were treated from the October 2020 to June 2021 with baseline demographics shown in Table 1. The median age was 68 (range 60−80). No patients received prior or concurrent Androgen Deprivation Therapy (ADT).

**TABLE 1:** Baseline patient demographics.

	Patient 1	Patient 2	Patient 3	Patient 4	Patient 5	Patient 6	Patient 7
Age	70	60	70	66	80	71	65
Gleason score at prostatectomy	4 + 3	4 + 3	4 + 3	4 + 3	3 + 4	4 + 3	3 + 4
Pre‐treatment PSA (ng/mL)	0.38	0.25	0.2	0.22	8.1	0.15	0.22

Treatment was well tolerated with 234 fractions treated on the MR Linac using the ATS protocol. Three patients required four fractions each (12 fractions in total) to be delivered on a standard linear accelerator due to machine issues.

### 3.1 Plan aim analysis

**FIGURE 5:**
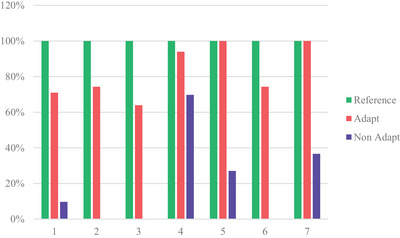
CTV coverage plan satisfaction.

Pre‐treatment reference plans met all CTV and OAR criteria. The percentage of online fractions that met criteria is laid out in Figure 5. Dosimetric Criteria for target volume coverage is laid out in Supplementary material (See Appendix 1, Supplementary materials).

### 3.2 Interfraction variation

Target volume and OAR volumes varied across fractions (Table 2). Across all patients, the CTV ranged from 64.3–257.2 cc, and varied 61%–141% when compared to the reference volume. Variation in OAR volume was more marked. Rectum volumes ranged from 22 cc to 274 cc; varied 58% to 214% of the reference volume. Bladder volumes ranged from 18.7 to 779.6 cc; varying from 23% to 197% of the reference volume. While large variations were seen, overall volume variation throughout all fractions was low. The average CTV volume across all fractions for all patients only varied by 1% from the average reference CTV volume, 7% for rectum volume and 12% for bladder volume.

**TABLE 2:** Reference and adapted contour volumes.

Volume (cm^3^)	Patient 1	Patient 2	Patient 3	Patient 4	Patient 5	Patient 6	Patient 7
Reference CTV	122.5	74.8	138.6	115.9	60.2	90.4	194.0
Average CTV (Range)	110.3 (88.4–141.3)	80.4 (64.3–115.6)	115.6 (85.3–160.3)	107.4 (90.5–186.0)	52.9 (27.6–99.2)	96.4 (68.2–119.4)	191.5 (124.5–257.2)
Reference rectum	58.3	74.4	152.7	46.5	57.5	68.3	42.6
Average rectum	40.0 (22.2–107.3)	34.5 (30.0–48.2)	108.1 (73.9–258.0)	61.1 (22.8–274.5)	53.1 (22.8–86.0)	114.1 (63.7–213.8)	42.3 (32.5–50.0)
Reference bladder	220.7	152.0	293.0	378.1	167.1	368.3	342.0
Average bladder (Range)	137.8 (35.9–294.2)	210.2 (82.5–484.5)	265.9 (18.7–533.0)	162.8 (31.9–443.0)	197.2 (46.6–493.9)	420.2 (105.0–779.6)	204.5 (66.2–462.7)

### 3.3 Treatment time

The treatment time from setup through completion of beam on time was an average of 36 min. A breakdown is shown in Table 3. Timing breakdown was available for 189 clinically delivered fractions.

**TABLE 3:** Treatment time breakdown (minutes).

	Setup	Imaging	Image review	Registration	Contouring	ED check	Optimization	RadCalc check	Beam on	Total
Mean	2.61	3.98	2.44	2.10	10.23	0.06	6.33	2.00	6.20	35.89
Standard deviation	0.60	2.34	0.97	0.90	6.20	0.31	2.51	0.95	1.73	8.49

### 3.4 Toxicity

At three months, only two patients had treatment related toxicities. One patient experienced Grade 2 gastrointestinal toxicity, in the form of increased bowel frequency from pre‐treatment baseline of two bowel motions per day to 3–4 per day. This had normalized at the time of their 6‐month post treatment review. A separate patient experienced Grade 1 genitourinary toxicity in the form of increased nocturia on top of their pre‐treatment baseline.

At 6 months, only one patient had treatment related toxicity. This patient, who had previously experienced Grade 2 gastrointestinal toxicity, had improvement in symptoms to Grade 1.

### 3.5 DICE similarity

DICE Similarity was calculated for PTV, bladder and rectum to assess the accuracy of the deformable image registration. The mean dice coefficient for PTV ranged from 74%–86%, for bladder 75%–91%, for rectum 75%–82%.

### 3.6 Dose comparison of different workflows

**FIGURE 6:**
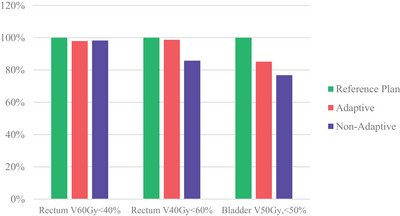
OAR dose constraint satisfaction.

The percentage of plans that met CTV coverage constraints was higher in the clinically delivered adaptive plans compared to the non‐adaptive plan comparison (82.48% vs. 20.44%; see Figure 5). PTV coverage satisfaction was also far higher in the adaptive plans than non‐adaptive plans; 42.93% versus 7.27% respectively. Organ at Risk constraints were consistently met in the adaptive plans (Figure 6).

## 4 DISCUSSION

Salvage prostate bed radiotherapy is an important and often underutilized treatment in men with biochemically recurrent prostate cancer following radical prostatectomy.^24^ We successfully implemented a fully adapted online treatment program on a 1.5 T MR Linac.

In the case of post‐prostatectomy radiotherapy, the prostate bed volume is not consistent throughout a treatment course as it is dependent on the position of the bladder and rectum, which can differ daily as seen in our reported variation in volume size. Given the wide variability in CTV and OAR position and volumes outlined above, we believe that this makes it an ideal tumor site for MR adaptive treatment. CTV coverage can be optimized, and OAR sparing can be tailored to the individual during each fraction to mitigate the chance of geographic miss as well as reduce dose to surrounding normal tissues prone to troublesome acute and late toxicity.

A key factor in developing this treatment technique was the time taken for treatment delivery given the intrafraction bladder filling. Changes in bladder filling can affect position of the anterior CTV margin as well as patient comfort. We chose to use an ATS workflow given the changes occurring daily. This required daily contour adaption, which comprised the largest portion of treatment time. As time progressed, contouring and overall treatment times decreased with each patient, reflecting the increase in experience in treating this subsite. All patients tolerated treatment well, with no patients requiring an interruption of their fraction due to bladder or other discomfort. Following radiation oncologist review, slight underdosage to the CTV to ensure on‐bed time and patient comfort was maintained. Based on the volume variation and the proximity of the limiting OARs clinical coverage in some cases was accepted to maintain OAR dose limits. In this setting the daily anatomy may make reference planning constraints unachievable, even when adapting. Presumably, if this was investigated for non MRL CBCT guided treatments the result would be similar. Furthermore, quantitative assessment on the difference in volume and dosage between T0 and T25 would be beneficial to aid clinical decision making, with the possibility of future proprietary and experimental tools to assist with this process.

Whilst a mix of ATP and ATS is often utilized in the implementation of MRgART, for long course treatments, the study of the use of a fully adaptive ATS workflow allowed acquisition of full image data sets for subsequent comparison of different adaptation strategies. A total of 234 daily adaptive plans were clinically delivered with a further 234 non‐adaptive plans generated to allow dose accumulation analysis and examination of the adaptive workflow. Comparing each clinically delivered adaptive plan with an individually generated surrogate plan of a traditional non‐adaptive treatment enabled comparison on the impact adaptive radiotherapy has in situations with interfraction variation in target volume and OAR volumes. Based on the dose accumulation comparison, the clinically adapted radiotherapy plans met dosimetric criteria more often than their non‐adapted comparators.

Early experience suggests that reported toxicity is low, consistent with similar studies of MRgART to this anatomical site.^10^ Further research may determine whether this is due to the ability for adaptive treatment to provide excellent target volume coverage whilst minimizing dose to adjacent organs at risk. When analyzing the utility of a novel treatment technique, it is paramount to compare it to current treatment standards. Further research is being undertaken to analyze the difference in target volume and organ at risk coverage for adaptive plans with previously standard of care non‐adaptive plans. Utilizing the reference plan and daily MRs acquired during the adaptive plan delivery process, dosimetric analysis on potentially delivered plans could allow for comparison between adaptive and non‐adaptive approaches. It may then be possible to establish a possible difference in dose coverage and OAR sparing between the two treatment modalities.

Other early studies have explored the use and questioned the benefit of adaptive radiotherapy in intact prostate radiotherapy. However, the variability in individual patient anatomy and dependence in part on the bladder as a basis of its treatment volume whilst also remaining and important OAR lends itself well to adaptive treatment to best adjust to daily change.

It is important to acknowledge the challenges with newer techniques in establishing robust evidence‐based frameworks for their use. This study is limited by its small patient numbers as well as short clinical follow‐up, but serves to lay the groundwork for further investigations into the broader applicability and use of the MR Linac and adaptive treatment in the pelvis. Owing to the difficulty in organ and tumor soft tissue delineation on standard X‐Ray based image verification systems as well as the highly variable nature of pelvic contents, MR‐based adaptive treatment may be of clinical benefit in gynecological, gastrointestinal and other genitourinary tumors within the pelvis.

Given the excellent clinician reported toxicities outlined, traditionally accepted OAR constraints may need to be re‐evaluated. Traditional OAR constraints which are derived from standard non‐adaptive treatment may be superseded by those based on dose volume histograms acquired from analysis of adaptive treatment. Isotoxic treatment, enabling personalized treatment considering variability in target volume and that of adjacent organs could allow for treatment to higher dose. Furthermore, future research may allow consensus‐based updates on PTV margins with the advent of MRgART. This may in turn enable higher local control rates with clinically minimized or comparable toxicity rates.

## 5 CONCLUSION

Daily MRgART treatment consistently met planning criteria. Target volume variability in prostate bed treatment can be mitigated by using MRgART and deliver satisfactory coverage of CTV whilst minimizing dose to adjacent OARs and reducing toxicity.

## AUTHOR CONTRIBUTIONS

Sean P. Hassan contributed to scientific design, data acquisition, data analysis and drafted manuscript. Jeremiah de Leon contributed to scientific design, data acquisition, data analysis and manuscript review. Vikneswary Batumalai contributed to scientific design, data acquisition, data analysis and manuscript review. Zoe Moutrie contributed to scientific design, data acquisition, data analysis and manuscript review. Yuanyuan Ge contributed to scientific design, data acquisition, data analysis and manuscript review. Phillip Stricker contributed to scientific design, data acquisition, data analysis and manuscript review. Michael G. Jameson contributed to scientific design, data acquisition, data analysis and manuscript review.

## CONFLICT OF INTEREST STATEMENT

GenesisCare has institutional research agreements with Elekta AB and ViewRay Technologies Inc. MGJ declares speaker honoraria and travel from Elekta AB and licensing agreement with Standard Imaging Inc. JdL declares speaker honoraria and travel from Elekta AB.

## Supporting information

Supporting information
