# Supplementary material for: Magnetic resonance guided adaptive post prostatectomy radiotherapy: Accumulated dose comparison of different workflows
Source: J Appl Clin Med Phys. 2024 Feb 23;25(4):e14253. doi: 10.1002/acm2.14253 (PMC11005979; doi:10.1002/acm2.14253)
Supplement: Supplementary file 1 — Supporting information [file ACM2-25-e14253-s001.docx]

Appendix 1: Individual Patient Target Volume Dosimetric Criteria

Patient 1

- CTV D98%>7000cGy
- CTV D99%>7000cGy
- PTV D98%>6650cGy

Patient 2

- PTV D98 > 6650cGy

Patient 3

- CTV D98%>7000cGy
- CTV D99%>7000cGy
- PTV D98%>6650cGy

Patient 4

- CTV D98%>7000cGy
- CTV D99%>7000cGy
- PTV D98%>6650cGy

Patient 5

- CTV D98%>7000cGy
- PTV D98%>6650cGy

Patient 6

- CTV D99%>6800cGy
- PTV D95>6664cGy

Patient 7

- CTV D98%>7000cGy
- PTV D98%>6650cGy
